# Proteomic Profiling and Biomarker Discovery in Colorectal Liver Metastases

**DOI:** 10.3390/ijms23116091

**Published:** 2022-05-29

**Authors:** Geoffrey Yuet Mun Wong, Connie Diakos, Thomas J. Hugh, Mark P. Molloy

**Affiliations:** 1 Department of Upper Gastrointestinal Surgery, Royal North Shore Hospital, Sydney, NSW 2065, Australia; tom.hugh@sydney.edu.au; 2 Northern Clinical School, The University of Sydney, Sydney, NSW 2065, Australia; connie.diakos@sydney.edu.au; 3 Department of Medical Oncology, Royal North Shore Hospital, Sydney, NSW 2065, Australia; 4 Bowel Cancer and Biomarker Research Laboratory, Faculty of Medicine and Health, School of Medical Sciences, The University of Sydney, Sydney, NSW 2006, Australia; m.molloy@sydney.edu.au

**Keywords:** colorectal cancer, colorectal liver metastases, proteomics, prognosis, biomarkers, mass spectrometry

## Abstract

Colorectal liver metastases (CRLM) are the leading cause of death among patients with metastatic colorectal cancer (CRC). As part of multimodal therapy, liver resection is the mainstay of curative-intent treatment for select patients with CRLM. However, effective treatment of CRLM remains challenging as recurrence occurs in most patients after liver resection. Proposed clinicopathologic factors for predicting recurrence are inconsistent and lose prognostic significance over time. The rapid development of next-generation sequencing technologies and decreasing DNA sequencing costs have accelerated the genomic profiling of various cancers. The characterisation of genomic alterations in CRC has significantly improved our understanding of its carcinogenesis. However, the functional context at the protein level has not been established for most of this genomic information. Furthermore, genomic alterations do not always result in predicted changes in the corresponding proteins and cancer phenotype, while post-transcriptional and post-translational regulation may alter synthesised protein levels, affecting phenotypes. More recent advancements in mass spectrometry-based technology enable accurate protein quantitation and comprehensive proteomic profiling of cancers. Several studies have explored proteomic biomarkers for predicting CRLM after oncologic resection of primary CRC and recurrence after curative-intent resection of CRLM. The current review aims to rationalise the proteomic complexity of CRC and explore the potential applications of proteomic biomarkers in CRLM.

## 1. Introduction

Globally, colorectal cancer (CRC) is the third most common cancer (10.0%) and the second leading cause of cancer death (9.4%) [1]. The liver is the most common site of CRC metastasis due to the portal venous drainage from the colon and rectum to the liver [2,3]. Colorectal liver metastases (CRLM) are detected in approximately 20% of patients at initial diagnosis and are the leading cause of death among patients with metastatic CRC [4,5,6]. Although CRLM portends a poor prognosis, liver resection is potentially curative in select patients, with actual 10-year recurrence-free survival reported in an estimated 20% of patients [7,8]. Multimodal treatment approaches have led to remarkable improvements in the prognosis of patients with CRLM over the past two decades. Although five-year overall survival rates after liver resection are as high as 50–60% in contemporary series, an estimated 75% of patients develop recurrence, and most occur within two years [9,10]. Whilst specific clinicopathologic variables are prognostic at baseline, conditional survival analysis in patients with resected CRLM demonstrates that these preoperative factors are inconsistent and lose prognostic significance over a relatively short time [11,12,13]. Early recurrence is the most useful single prognostic and clinical feature in estimating disease-specific survival, but the ability to predict this is currently limited [13,14,15]. Tumour genetics, location and treatment effect heterogeneity give rise to challenges in selecting treatment and predicting whether an individual might benefit from a particular treatment [16,17,18].

Patients with resectable CRLM require a nuanced approach given the expanding criteria of resectability and increasing treatment options [19,20]. This group presents a unique opportunity to understand the molecular underpinnings of metastatic CRC because they are free of detectable metastasis at a defined time point. The rapid development of next-generation sequencing technologies and the declining cost of human genome sequencing has accelerated genomic profiling of various cancers, including CRC [21,22]. Although the characterisation of genomic alterations in CRC has significantly improved our understanding of its carcinogenesis, the functional context at the protein level has not been established for most of this genomic information [23,24,25]. Starting from the genome, multiple biological regulatory and processing steps take place to arrive at the proteome, each step driving increasing complexity and diversity. Consequently, chemical modifications that affect protein function and protein–protein interactions that carry out critical biological activities cannot reliably be predicted from genomic and transcriptomic analyses. Moreover, because mutations do not always result in a predicted change in the corresponding proteins and phenotype, the gap between gene expression and the biological capability of cancer is not straightforward [26,27,28,29]. Therefore, precision oncology requires the examination of the co-expression of multiple genes and proteins under different disease states and the impact these have on clinically meaningful outcomes such as recurrence and survival.

Liquid chromatography–mass spectrometry (LC-MS) of digested proteins conducted with high-resolution instruments allows us to quantitate thousands of proteins from complex biological specimens in either data-dependent acquisition, or more recently, data-independent acquisition workflows [30,31]. Preclinical exploratory studies on proteomic profiling of cancer biospecimens have provided new insights into the molecular alterations in cancer and have identified leads for potentially useful clinical biomarkers. Proteomic mass spectrometry data have often accompanied landmark cancer genomic studies; for example, those reported on colorectal cancer, pancreatic cancer, ovarian cancer and lung cancer [32,33,34,35,36]. Publications have increased steadily in mass-spectrometry-driven proteomics analysis of differential protein expression and cancer-specific biomarkers derived from tissue and body fluids. Several studies have explored proteomic biomarkers in predicting CRLM after oncologic resection of primary CRC and recurrence after curative-intent resection of CRLM; however, there is no up-to-date overview of these findings. This review aims to rationalise the proteomic complexity of CRC and explore the potential applications of proteomic biomarkers in CRLM by critically appraising mass-spectrometry-based proteomic profiling of human CRLM biospecimens published over the last ten years. 

## 2. Characteristics of Preclinical Exploratory Studies on Proteomic Biomarkers in Colorectal Liver Metastases

Seventeen exploratory studies on proteomic profiling and five studies on proteogenomic profiling of human CRLM were identified through a search of the literature using PubMed, Medline and ScienceDirect, with the main search terms including “colorectal”, “cancer”, “liver”, metastasis” or “metastases”, “proteomics” or “proteome”, “proteogenomics”, “biomarker”, “mass spectrometry” and “prognosis”. Relevant studies on human biospecimens from 2011 to 2021 were included and studies that focused on animal models, cell lines and patient-derived xenograft models were excluded. References contained in the included studies were reviewed for appropriate publications that the electronic search strategy may have missed. Proteomic and proteogenomic studies included are summarised separately in Table 1 and Table 2, with the most recent publications listed first. The studies included samples from 301 patients with CRLM, with a range of 1–44 patients in each exploratory cohort. New proteomic signatures were revealed even in studies with small sample sizes, and therefore these were included. Sixteen studies used fresh frozen tissue and six used formalin-fixed paraffin-embedded (FFPE) tissue. Although only studies that reported MS-based proteomics were selected and the majority utilised LC-MS, there were variations and nuances in the techniques utilised across studies. Most of the included studies performed differential protein expression analysis to quantify protein abundance between two or more groups within the same experiment. The comparison groups included a combination of matched or unmatched primary CRC, normal colonic tissue, normal liver tissue, or prognostically different patient groups stratified by clinicopathological factors. 

## 3. Proteomic Profiling of Colorectal Liver Metastases Tissue Identifies Prognostically Distinct Groups

The clinical utility of prognostic prediction models in CRLM has been limited, as these scoring systems do not consistently stratify recurrence and survival after curative-intent surgery [13,59]. Clinical risk-scoring systems were marginally better than chance alone in predicting outcomes in some cohorts [12]. Prognostic biomarkers indicate the likelihood of a future clinical event, disease recurrence, or disease progression among patients with the same characteristics [60,61]. One existing strategy to overcome the current limitations of clinical risk scores is to identify prognostic biomarkers that indicate the likelihood of recurrence after resection of CRLM. Early recurrence is associated with poor prognosis and is a useful clinical feature in estimating conditional disease-specific survival [14,15,62]. 

Michal et al. characterised proteomic biomarkers in prognostically distinct clinical groups based on the time interval between the resection of CRLM and recurrence. A 12-month cut-off was used to divide patients into those with a ‘good prognosis’ (*n* = 29) and ‘poor prognosis’ (*n* = 29). Microdissection of FFPE tissue followed by label-free LC-MS identified 99 differentially expressed proteins, of which a third were associated with the extracellular matrix pathway. MMP7 and DPEP1 were upregulated, while LOXL1 was downregulated. MMP7 promotes invasion through proteolysis of the ECM proteins and proliferation of cancer cells through upregulation of MM2 and MMP9. In addition, MSH2 and MCM4, associated with DNA replication and repair pathways, were upregulated, and several components of the immune pathway—such as C5, C1RL, C8A, CD163, chymase 1, and HLA-B—were downregulated in the poor-prognosis group. This study indicated that components of the tumour microenvironment, especially the extracellular matrix pathway, may be critical drivers in early recurrence after resection of CRLM [37].

The study by Snoeren et al. used gene expression profiling and label-free nano-LC-MS/MS to identify genes and proteins that correlate with early (<6 months) and late (>24 months) recurrence after resection of CRLM. Upregulation of SERPINB5 and increased expression of Maspin were the only overlapping factor among 14 genes and 46 proteins that showed a significant association with recurrence. Immunohistochemical analysis of Maspin expression in stage III CRC correlated with early time to recurrence and disease-specific survival, but not in stage II CRC. Altogether, these findings point to Maspin as a potential biomarker for early recurrence in primary stage III and IV colorectal cancer [58].

## 4. Adjuvant Treatment Stratification for Stage II and Stage III Colorectal Cancer

Following oncologic resection of primary CRC, approximately 20% of patients with stage II and 30–40% of patients with stage III colorectal cancers will develop recurrence [63,64,65]. Follow-up after oncologic resection for primary colorectal cancer includes regular monitoring of carcinoembryonic antigen (CEA) and cross-sectional imaging to ultimately increase patient survival rates and quality of life through the early detection of recurrent disease. Adjuvant chemotherapy aims to eradicate cancer micrometastases. However, most patients with stage II CRC (i.e., those without regional lymph node metastasis) undergo clinical surveillance following oncologic resection of the primary tumour as the benefit of adjuvant chemotherapy has not been demonstrated in low-risk stage II CRC. Several high-risk clinicopathological features for recurrence have been identified but there is no clear evidence of patient selection and limited evidence on the benefit of adjuvant chemotherapy in this situation [66,67,68]. Therefore, the rationale for proteomic profiling in this patient group is to identify those at higher risk of recurrence, as these patients may benefit from adjuvant chemotherapy or more intensive surveillance. Kirana et al. used a combination of laser microdissection of primary CRC, two-dimensional differential gel electrophoresis (2D-DIGE) and matrix-assisted laser desorption ionisation time-of-flight mass spectrometry (MALDI-TOF MS) to identify protein biomarkers that stratify the risk of CRLM in patients with stage II disease. Cancer cells from patients who developed recurrence (*n* = 11) and those who did not develop recurrence (*n* = 8) within five years of surgery were isolated using laser microdissection to minimise protein contamination from non-tumour tissue. A total of 55 differentially expressed proteins were identified by 2D-DIGE and MALDI-TOF MS. The expression of HLAB, ADAMTS2, LTBP3, JAG2, and NME2 was among ten differentially expressed proteins significantly associated with vascular invasion and CRLM. These prognostic protein biomarkers may be useful in complementing current cancer staging systems and predicting the risk of CRLM in stage II CRC [47].

Adjuvant chemotherapy with fluoropyrimidine combined with oxaliplatin has been the standard of care for stage III CRC patients with good performance status and who can tolerate cytotoxic combination chemotherapy [69,70]. Although the therapeutic indication for this patient group is significantly less controversial than for patients with stage II CRC, the optimal duration of adjuvant chemotherapy is unclear [71]. The absolute difference of 0.9% in 3-year disease-free survival between patients receiving six versus three months of adjuvant chemotherapy is associated with increased toxicity and potential impairment of quality of life [72,73]. Yang et al. reported the protein expression profiles of tissue samples from patients with stage III and CRLM to identify key proteins related to progression in CRC. Protein expression profiles of patients with stage III CRC (*n* = 20) and CRLM (*n* = 17) were acquired using a label-free proteomics approach and nanoflow liquid chromatography coupled to an ultra-high-resolution mass spectrometer (nano-LC-MS/MS) [45]. Weighted correlation network analysis enabled clustering of co-expressed proteins into modules that correlated with traits [74]. Three modules were significantly correlated with CRC, from which nine proteins were identified through protein–protein interaction networks. Fibrinogen beta chain (FBG), Talin 1 (TLN1), and adaptor-related protein complex 2 subunit alpha 2 (AP2A2) all had a strong positive correlation with CRLM. HSPD1, EEF1G, and HNRNPA2B1 were positively correlated with primary CRC and CRLM. SRRT, APOC3, and PGM5 were key proteins associated with primary stage III CRC and provide insight into the progression from stage III CRC to CRLM [45].

## 5. Comparison of Colorectal Liver Metastases and Primary Colorectal Tumours

The molecular classification of colorectal cancers (CRCs) into intrinsic subtypes may be useful in refining prognosis and predicting patient outcomes [75,76]. Several studies have evaluated the proteome of primary CRC and matched colorectal liver metastases (CRLM) to establish the molecular basis of metastatic CRC [38,39,44,46,49]. Understanding the continuous evolution of CRC underscores the development of effective and targeted approaches across the spectrum of CRC [77].

A pilot study by Farhner et al. compared the proteome of seven matched FFPE specimens of primary CRC and CRLM using liquid chromatography–mass spectrometry (LC-MS/MS). Unsupervised clustering of over 2600 proteins demonstrated differences in the proteome of primary CRC and corresponding liver metastases. Many upregulated proteins in CRLM involve glucose metabolism, including pyruvate carboxylase, fructose-bisphosphate aldolase B and fructose-1,6-bisphosphatase 1. CRLM demonstrated an active immune response compared to primary CRC, as reflected by the upregulation of several complement system components, including C1, C4, C5 and C9. Multiple structural proteins associated with muscle contraction and cell junction assembly, such as desmin, synemin and filamin-C, were depleted in CRLM compared to primary CRC [38]. The molecular changes from primary CRC and CRLM highlight the distinct proteome of primary CRC and corresponding CRLM.

The proteomes of primary CRC and CRLM were compared by Liu et al. and Ku et al. using tandem mass tag (TMT)-labelling and LC-MS/MS [39,44]. TMTs are chemically reactive agents that impart isotope-based differences to peptide amines, enabling multiplexed LC-MS for peptide identification and simultaneous quantitation. TMT sample multiplexing facilitates high-throughput, large-scale quantitative proteomics data acquisition [78,79]. Liu et al. conducted a comparative analysis of proteomics between the primary CRC and CRLM in eight patients (*n* = 8). Several extracellular matrix components including FN1, TIMP1, THBS1, POSTN and VCAN, were upregulated in CRLM. Secondary analysis with immunohistochemistry revealed that increased THBS1 expression was significantly correlated with CRLM and poor prognosis. The role of THBS1 (Thrombospondin 1) in facilitating CRLM through enhancing epithelial–mesenchymal transition was supported by transwell cell migration and invasion assays, which in turn demonstrated that THBS1 depletion inhibited the migration and invasion of CRC cells [39]. Ku et al. used TMT labelling with LC-MS to compare of the proteomic profiles of fresh frozen tissue from nine patients (*n* = 9) and demonstrated protein signatures that distinguished CRLM and its primary and normal colon tissues. In total, 47 differentially expressed proteins were statistically significant between primary CRC and CRLM, of which Filamin A-interacting protein 1-like (FILIP1L) and plasminogen (PLG) were novel signature proteins described in CRLM. FILIP1L has been shown to suppress tumour progression by inhibiting cell proliferation and angiogenesis in CRC; hence, underexpression may contribute to CRC metastasis. Plasminogen, which showed significantly high expression, may allow for tumour attachment and invasion through the basement membrane and is associated with a worse CRC prognosis [44].

Synchronous CRLM are present in 15–25% at the index presentation with CRC [6]. The management of synchronous CRLM is more complex than metachronous CRLM and the prognosis for these patients is worse [80,81]. Kim et al. utilised an approach that combined 2D polyacrylamide gel electrophoresis and MALDI-TOF MS to identify metastasis-related factors differentially expressed in primary CRC and CRLM. The study identified 58 differentially expressed proteins between primary CRC and synchronous CRLM. Seven differentially abundant proteins were upregulated: SERPINA1, APOA1, ITLN1, DES, DBI, SDHA and CA1. Compared to primary CRC, pertinent biological processes altered in CRLM included increased energy metabolism and decreased immune-cell-related migration. The location of these differentially expressed proteins in the extracellular region and exosome or membrane-bound vesicles make these potentially useful circulating biomarkers [46].

Autoantibodies are produced by an immunological response to cancer cells [82]. Yang et al. used an immune-proteomic strategy to discover tumour tissue autoantigens from eight matched primary CRC, CRLM and adjacent normal liver tissue. Antigens from paired CRLM and normal liver tissue were identified using serum from patients with autoimmune disease. Furthermore, 1D and 2D gel electrophoresis and Western blotting were used to detect reactive protein bands, then these were analysed using mass spectrometry. Overall, 48 proteins were uniquely found in CRLM and absent in normal liver tissue. Olfactomedin 4 (OLFM4), CD11b, integrin α2 (ITGA2), periostin and thrombospondin-2 were reproducibly identified on Western blotting and mass spectrometry. These antigens were also overexpressed in primary CRC. OLFM4, CD11b and ITGA2 were validated in two cohorts [49]. OLFM4 is an anti-apoptotic factor and colon stem cell marker, whereas CD11b and ITGA2 are integrins that have a recognised role in promoting epithelial–mesenchymal transition and metastasis in CRC [83,84,85]. The concordant overexpression of these three biomarkers in both primary CRC and CRLM may be helpful in predicting the risk of CRLM and inform the development of immunotherapy for the treatment of CRC.

## 6. In-Depth Proteomic Characterisation of Colorectal Liver Metastases

Tumour heterogeneity describes differences between cancer cells within a tumour and leads to challenges in precision oncology. Genomic instability is a significant cause of genetic heterogeneity, a genetic feature of adenomatous tumours [86,87]. Although similar changes would be expected at the protein level, Turtoi et al. rationalised the proteome heterogeneity in CRLM by demonstrating a distinct and organised pattern of molecular alterations. Matrix-assisted laser desorption ionisation (MALDI)–mass spectrometry-based imaging and in-depth proteomic analysis of eight fresh CRLM samples and their corresponding normal tissue showed a reproducible, zonally delineated spatial distribution of over 1000 proteins. The centre of the lesion was characterised by elevated carbohydrate metabolism and DNA-repair activity, the rim of the metastasis displayed increased cellular growth movement and drug metabolism, and the peritumoral region featured elevated lipid metabolism and protein synthesis. LTBP2 and TGFB1 were two novel antigens consistently expressed in CRLM and were amenable to antibody-based tumour targeting in vivo, highlighting their therapeutic potential [52]. 

Progressive alterations in the proteome characterised by different extracellular matrix phenotypes have been reported in a single patient with metachronous CRLM and three curative-intent hepatic resections. Proteome analysis using LC-MS/MS identified 481 differentially regulated proteins, 81 of which were associated with the extracellular matrix and previously reported as negative prognostic markers, including tenascin C, nidogen 1, fibulin 1 and vitronectin. The clinical and proteomic findings correlate with increasing metastatic potential with each subsequent recurrence and support the rationale for comprehensive molecular analysis of metastases from different time points during disease progression [40].

## 7. Proteomic Profiling of the Extracellular Matrix in Colorectal Liver Metastases

The extracellular matrix (ECM) is a major component of the tumour microenvironment and comprises a complex network of macromolecules, such as proteins and polysaccharides secreted locally by cells [88]. In addition to its commonly recognised function of providing cells with structural support and mechanical integrity, the ECM is involved in biochemical signalling that modulates the hallmarks of cancer [89,90]. Cancer-associated ECM plays a significant role in sustaining proliferative signalling, evading growth suppressors, resisting cell death, enabling replicative immortality, inducing angiogenesis, as well as activating invasion and metastasis. ECM has also been implicated in emerging cancer hallmarks, including avoiding immune destruction, dysregulating cellular energetics, promoting genomic mutation and instability, and modulating immune cell behaviour and inflammation [89,90]. The abundance of protein in the ECM and its complex role in tumorigenesis have been the focus of several studies on proteomic profiling of the ECM in colorectal liver metastases (CRLM) [41,42,43,51].

A three-part series of mass spectrometry-based studies by van Huizen et al. on the role of collagen and its posttranslational modifications in CRLM have provided a deeper understanding of ECM’s role in CRLM tumour biology [41,42,43]. The authors first demonstrated that specific collagen proteins were upregulated in CRLM compared to adjacent normal liver tissue in 30 FFPE CRLM samples. Out of 22 collagen-α chains, 19 were significantly (*p* < 0.05) upregulated in CRLM. The upregulation of 16 proteins required for collagen synthesis further supported increased collagen synthesis in metastatic CRC. Further, six non-collagen proteins (CDH17, KRT20, CEACAM5, GPA33, MUC13, and PPP1R1B/DARPP-32) were upregulated in CRLM, where CHD17 and PPP1R1B/DARPP-32 have not been described previously [43]. A subsequent study comparing CRLM and adjacent normal tissue (*n* = 2) identified posttranslational modification by enzymatic hydroxylation of proline at the Xaa position in collagen. Here, reduced 4-hydroxyproline in CRLM clearly distinguished it from control liver tissue [42]. Validation of these findings using a reference to a synthetic standard peptide in a larger sample (*n* = 14) showed consistent down-regulation of collagen hydroxylation in CRLM. Furthermore, the degree of hydroxylation of control liver and colonic tissue were similar, differentiating CRLM based on these posttranslational modifications [41]. Altogether, the differences in collagen types in CRLM may reflect altered collagen stability and could serve as potential prognostic biomarkers.

Naba et al. characterised the ECM composition of matched primary CRC, CRLM, and normal colonic tissue (*n* = 3) using ECM enrichment and liquid chromatography-tandem mass spectrometry [51]. The properties of ECM proteins, such as their large size, cross-linked and covalent bonds, and heavy glycosylation, render them challenging to analyse. The subcellular fractionation protocol described by Naba et al. takes advantage of the insolubility of ECM proteins to preferentially remove cytosolic proteins, nuclear proteins, membrane proteins, and cytoskeletal proteins, leaving a final insoluble fraction enriched for ECM. The ECM-enriched protein preparations are then digested into peptides for subsequent MS analysis [91,92]. Using these methods, robust signatures of ECM proteins that characterised each tissue were defined, amongst which COMP, FNDC1, IGFALS, SPP1, BMP1, CIQTNF5, and HPX were characteristic for CRLM. The ECM composition of CRLM and primary CRC showed a closer resemblance than normal liver tissue, with 23 proteins shared by primary CRC and CRLM. EGF-containing fibulin-like ECM protein 2 or Fibulin 4, thrombospondin 2, and tissue inhibitor of metalloproteinase-1, which were detected in both primary and secondary colon tumour tissue—but not in healthy tissue—have been shown to be detectable in serum of patients with CRC [93,94,95]. The studies to date point to a crucial contribution of the extracellular matrix. The dynamic range of ECM proteins may prove to be valuable indicators of progression or recurrence in CRC but warrant further validation. 

## 8. Post-Translational Protein Modification in Colorectal Liver Metastases

Post-translational modification of cancer-associated extracellular matrix (ECM) alters the interaction of cancer cells with its microenvironment and influences malignancy and tumour growth [96]. Citrullination is produced through post-translational deamination of peptidyl-arginine and is catalysed by peptidylarginine deiminase (PAD). PAD and citrullination have been implicated in cancer development through several mechanisms such as activation of cancer cell signalling, alteration of epithelial-to-mesenchymal transition, formation of neutrophil extracellular traps and induction of antitumour activity [97]. Yuzhalin et al. identified tumour-derived peptidylarginine deiminase 4 (PAD4)-driven citrullination of ECM proteins to be essential for CRLM growth. ECM-enrichment and quantitative label-free analysis of proteomic data identified 287 proteins with statistically significant abundance between CRLM and normal liver, of which 69 proteins were upregulated or downregulated within the ECM by more than 3-fold. Among the upregulated proteins, versican, TIMP1, LTBP1–3, DDR1, and S100A10 have been previously linked to metastasis. ECM proteins are highly citrullinated in CRLM compared to normal liver, primary CRC and normal colonic mucosa. The upregulation of PAD4, which was 11 times more abundant in the ECM of CRLM than in normal liver, was also specific to CRLM and likely accounts for the increased citrullination of proteins in CRLM. CRC cell lines showed greater adhesion when cultured on citrullinated collagen type I than on non-citrullinated control and an increased expression of epithelial markers. These findings suggest that citrullination confers metastatic properties to CRC cells through enhanced epithelial–mesenchymal transition. Additionally, the functional significance of these post-translational modifications was demonstrated in murine models, where inhibition of PAD4 activity reduced citrullination and CRLM growth [48].

Enzymes and proteins involved in acetylation, typically on lysine residues, regulate many cellular physiological processes but are deregulated in cancer [98]. These alterations may have functional implications in cancer biology, such as metabolic reprogramming and adaptation to the tumour microenvironment [99,100]. Global-scale profiling of differentially expressed lysine-acetylated proteins in matched primary CRC and CRLM was first reported by Shen et al. This study characterised the acetylome paired primary CRC and CRLM samples (*n* = 3) using tandem mass tag protein labelling, high-affinity enrichment of acetylated peptides and LC-MS/MS analysis. A total of 603 acetylation sites from 316 proteins were identified, and 462 acetylation sites corresponding to 243 proteins were quantified. Further, 31 acetylated sites of 22 proteins were downregulated, while 40 acetylated sites of 32 proteins were upregulated in CRLM. Among differentially expressed acetylated histone proteins between primary CRC and CRLM, acetylated histone H3.2 at Lys 19 (HIST2H3AK19Ac) showed the most significant downregulation. In contrast, acetylated histone H2B type 1-L at Lys 121 (H2BLK121Ac) was the most overexpressed acetylated histone in CRLM. TPM2 K152Ac was the most downregulated acetylated non-histone protein and ADH1B K331Ac was the most upregulated non-histone protein in CRLM. Most of the identified acetylated proteins were localised within the cytoplasm, associated with binding, and involved in multiple biological processes such as metabolic pathways, carbon metabolism and biosynthesis of amino acids. The findings in this study demonstrate that protein acetylation may be pivotal in biological processes that drive the development and progression of CRLM [50].

## 9. Proteomics as a Principal Component of Multiomics in Colorectal Liver Metastases

Multiomics provides an integrated biological analysis approach and enables a more comprehensive understanding of molecular changes across multiple levels of biology [101,102]. The downstream signalling effects of genomic alterations can be characterised and evaluated by including proteomic and post-translational modification data [103,104]. Table 2 summarises five studies that have layered various omics in addition to proteomics to understand how cell processes in CRC are connected and communicate with each other [54,55,56,57,58]. The use of fresh frozen CRLM biospecimens and nanoscale liquid chromatography coupled to tandem mass spectrometry (nano-LC-MS/MS) characterises the few multiomic studies on CRLM to date.

Multiomic analysis has demonstrated that primary CRC and CRLM are highly similar at the genetic but not at the proteomic or phosphoproteomic levels. Furthermore, proteomic profiling has identified biologically and prognostically distinct CRC subtypes that may be useful in risk stratification [54]. Altered peptides can be precisely quantified and potentially utilised to predict distinct somatic mutations such as KRAS G12V, even when these genes are expressed at low levels and despite protein diversity [55]. Correlation of survival analysis and multiomic data have also identified new leads for prognostic biomarkers [54,56,57,58]. However, the multiomics space in CRC, as with many other cancers, is still in the preclinical discovery phase. Therefore, differentially expressed proteins in these studies are hypothesis-generating but require further validation and development to close the gap between biology and translation. The integration of proteomics and post-translational modification data nevertheless represents a substantial advance over prior genomic studies of CRC and directs the way to improve the molecular characterisation of clinical cohorts.

## 10. Limitations in Proteomic Biomarker Discovery in Colorectal Liver Metastases

Proteomic biomarker discovery and development presents many biological, technical, and technological challenges [105,106]. Consequently, proteomic biomarkers in CRLM have not progressed beyond the discovery phase after more than a decade of intensive research, and none of the identified leads for potentially useful biomarkers has translated to clinical practice. Recognising the general and context-specific challenges in proteomic biomarker development in CRLM is essential to increasing value and reducing waste in the future [107].

The fundamental challenge with proteomic profiling in CRLM to date is the small sample size of existing studies [107,108]. Multiple biological processes occur from the genome to the proteome and result in many levels of protein diversity [109,110]. Such tremendous protein diversity favours the chance to discover lead biomarkers, even in studies with small sample sizes. As a result, there may be a strong reporting bias of statistically significant proteomic biomarkers in CRLM. Small-sample-size studies also reduce the chance of detecting a true effect and result in the overfitting of identified lead biomarkers. Clinical and cancer heterogeneity, both within and between these exploratory studies, further complicates the differentiation of candidate biomarkers from spurious results due to random variation [111]. The dynamic range of the cellular proteome and lack of direct amplification mechanisms, unlike in DNA, create technological challenges for identifying proteins at low concentrations that may significantly impact tumour biology, particularly from specimens preserved with formalin [112,113]. Most potential and novel biomarkers in CRLM have subsequently not been validated beyond the initial discovery study to determine their reproducibility and generalisability. Part of this problem lies with the academic endeavour, which rewards the detection of new markers in favour of large-scale studies that can be tedious and high-risk. Although several studies have been published on proteomic biomarkers in CRLM, data cannot be directly combined or compared due to the complexity of overlaying proteomic data from different platforms.

To increase the discovery of accurate biomarkers, the sample selection needs to be based on clearly defined and clinically meaningful endpoints with distinct prognostic implications, e.g., recurrence within six months (i.e., early recurrence) of resection of CRLM and 10-year actual recurrence-free survival. Proteomic biomarkers are most needed at decision nodes where the performance of prognostic or predictive factors is uncertain or inconsistent. Cohorts from which samples are selected need to be well characterised and closely matched to the specific clinical context to which these proteomic biomarkers are intended to be applied. However, simply conducting more siloed work will not address the current challenges in translational proteomics in CRLM. The complexities of proteomic biomarker development require a new level of collaboration to enable large-scale, unbiased, deep, proteomic profiling and subsequent validation to capture the true value of proteomics in improving clinical outcomes for patients with CRLM.

## 11. Translational Proteomics in Colorectal Liver Metastases

**Figure 1:**
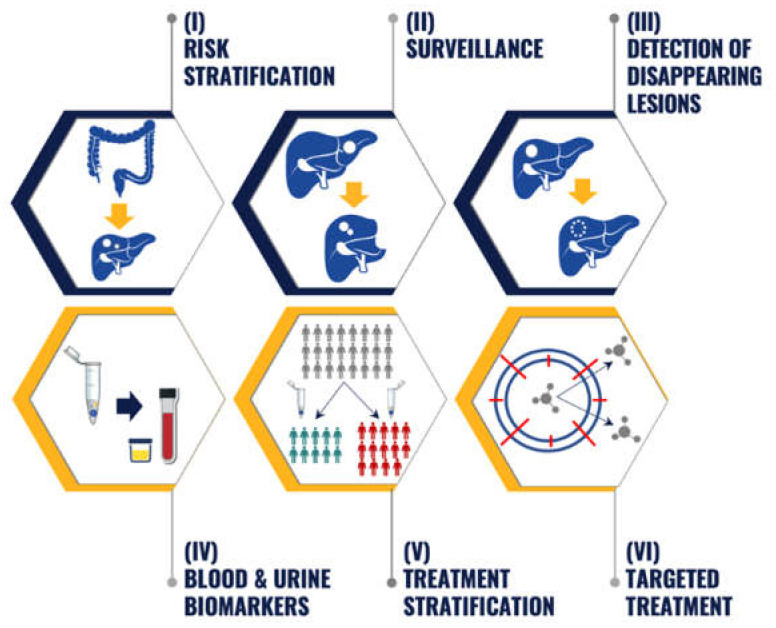
The potential clinical application of proteomic biomarkers in colorectal liver metastases (CRLM). (**I**) Risk stratification—assess the likelihood that CRLM will develop after oncologic resection of primary colorectal cancer (CRC). (**II**) Surveillance—early detection and treatment of recurrence after curative-intent resection of CRLM. (**III**) Detection of disappearing lesions—characterise the disease course and inform the surgical management of disappearing CRLM after preoperative systemic therapy. (**IV**) Blood and urine biomarkers—identify blood and urine biomarkers to monitor metastatic CRC progression and treatment. (**V**) Treatment stratification—predict response to multimodal therapy and select treatment that is most likely to yield a favourable response. (**VI**) Targeted treatment—patient selection for biomarker-driven clinical oncology trials.

Proteomic biomarker discovery and profiling reflect novel opportunities and approaches beyond genomics in managing patients with CRLM patients. The next step is to translate such proteomic discoveries into clinical applications (Figure 1). Practical applications include risk stratification, surveillance, detection of disappearing CRLM, monitoring cancer recurrence and treatment response through using blood/urine biomarkers, treatment stratification and development of targeted treatment. 

Differing proteomic profiles among patients with stage II/III CRC who subsequently develop metastatic disease or remain disease-free demonstrate the prognostic value of the proteomics-based applications for predicting recurrence after oncologic resection of the primary tumour [45,47]. Similarly, prognosis-based stratification can be determined through differing proteomic expression profiles following curative-intent CRLM resection [37,58]. Consequently, proteomic biomarkers can possibly be applied as part of a tailored surveillance and treatment strategy based on a patient’s molecular risk profile. Given the limitations of clinicopathologic predictors for CRLM, such potential CRLM proteomics-based applications represent additional analytical tools for molecular stratification.

The distinct CRLM and ECM proteome from normal hepatic tissue can serve as valuable proteomic targets for detecting post-chemotherapy disappearing CRLM [51,52]. The characterisation of differentially expressed proteins within tissue is also essential to identify candidate proteins for liquid biopsies. As most drugs target proteins, proteomic profiling of CRLM also has high value for drug discovery and development for metastatic CRC. The above-described studies offer a glance into the future of proteomics in CRLM, though further research efforts are required to translate these discoveries into clinical applications that improve individual CRC patient outcomes. 

## 12. Conclusions

This article critically reviewed the current state of emerging proteomic biomarkers using human CRC tissue specimens and mass spectrometry-based techniques. Variations and nuances in mass spectrometry-based approaches and proteomic analysis highlight the breadth of customisable methods to study the proteome in CRC. The diversity of proteomic profiles identified to date reflects cancer heterogeneity at different stages in the disease course of CRC. While multimodal cancer therapy advancements have significantly expanded curative-intent therapy in CRLM, some patients only derive limited benefits from current clinical approaches. The comprehensive characterisation of proteins in both primary CRC and CRLM has provided insight into similarities and continuations in molecular alterations, which form the basis for developing new biomarkers, targeted treatments, and therapeutic strategies. The preclinical exploratory phase of proteomic biomarkers has identified promising directions for future research. Coordinated research efforts are needed to streamline and optimise critical workflow steps to enable reproducible and accurate protein quantitation in specific clinical contexts and to unravel the complex mechanistic biology of CRC.

## Figures and Tables

**Table 1:** Studies on the prognostic relevance of mass spectrometry-based proteomic biomarkers in human colorectal cancer liver metastases over the last 10 years in descending chronological order.

First Author/ Reference/Year/Journal	Biospecimen	Mass-Spectrometry-Based Technique	Discovery Cohort Characteristics (Sample Size and Comparator)	Key Biomarkers and Findings
Michal S et al. [37] 2021 *Journal of Personalized Medicine*	FFPE tissue	Label-free LC-MS/MS	*n* = 29 with recurrence < 6 months after resection of CLRM Comparison: *n* = 29 with recurrence 6–12 months after resection of CRLM	Upregulation of matrix metalloproteinase 7 (MMP7) and dehydropeptidase 1 (DPEP1) in poor-prognosis group. Downregulation of lysyl oxidase-like 1 (LOXL1) in poor-prognosis group. A third of differentially expressed proteins associated with extracellular matrix.
Fahrner M et al. [38] 2021 *Neoplasia*	FFPE tissue	Label-free LC-MS/MS	*n* = 7 synchronous CRLM Comparison: *n* = 7 matched primary CRC	Metabolic proteins: pyruvate carboxylase (PC) and fructose-bisphosphate aldolase B (ALDOB), and fructose-1,6-bisphosphatase 1 (FBP1) upregulated in CRLM. Immune system proteins: enrichment of complement components C1, C4, C5, C9 in CRLM. Structural proteins: depletion of desmin (DES), synemin (SYNM) and filamin-C (FLNC) in CRLM.
Liu X et al. [39] 2020 *Clinical and Translational Oncology*	Fresh frozen tissue	TMT-labelling, LC-MS/MS	*n* = 8 CRLM Comparison: *n* = 8 primary tumour	Upregulation of fibronectin (FN1), metalloproteinase inhibitor 1 (TIMP1), thrombospondin-1 (THBS1), periostin (POSTN) and in CRLM.
Voß H et al. [40] 2020 *Clinical and Experimental Metastasis*	Fresh frozen tissue	Label-free LC-MS/MS	*n* = 1 with 3 metachronous CRLM Comparison: N/A	Upregulation of 56 extracellular matrix-associated proteins including tenascin C (TNC), nidogen-1 (NID1), fibulin-1 (FBLN1), vitronectin (VTN).
van Huizen NA [41] 2020. *Frontiers in Oncology*	FFPE tissue	Label-free nano-LC-MS/MS	*n* = 14 CRLM Comparison: *n* = 14 matched liver tissue, matched primary CRC and normal colonic tissue	Overall degree of collagen hydroxylation was significantly lower in CRLM and primary CRC compared to normal colon Downregulation of 11 peptides with a specific number of hydroxylation in CRLM compared to normal liver tissue.
van Huizen et al. [42] 2019 *Journal of Proteome Research*	FFPE tissue	Nano-LC-ESI-ETD-HCD	*n* = 2 CLRM Comparison: *n* = 2 matched normal liver tissue	Lower ratio of 4xHyp at position 584 of collagen alpha-2(I) chain (COL1A2) in CRLM.
van Huizen NA [43] 2019 *Journal of Biological Chemistry*	FFPE tissue	Label-free nano-LC-MS/MS	*n* = 30 patients Comparison: *n* = 30 matched normal liver tissue, primary CRC and normal colon tissue	Upregulation of four collagen types in CRLM: COL10A1, COL12A1 (most abundant), COL14A1, COL15A1. Upregulation of six non-collagen colon-specific proteins in CRLM: cadherin-17 (CDH17), protein phosphatase 1 regulatory subunit 1B (PPP1R1B/DARP-32), keratin, type 1 cytoskeletal 20 (KRT20), carcinoembryonic antigen-related cell-adhesion molecule 5 (CEACAM5), cell-surface AA33 antigen (GPA33), mucin-13 (MUC13).
Ku X et al. [44] 2019 *Analytical Cellular Pathology*	Fresh frozen tissue	TMT labelling, nano-LC-MS/MS	*n* = 9 CRLM Comparison: *n* = 9 matched primary tumour and normal colonic tissue	Upregulation of filamin A-interacting protein 1-like (FILIP1L) and plasminogen (PLG) in CRLM.
Yang W et al. [45] 2019 *Proteomics Clinical Applications*	Fresh frozen tissue	Label-free nano-LC-MS/MS	*n* = 17 CRLM Comparison: *n* = 20 Stage III CRC who did not develop CRLM	Nine key proteins identified in CRLM: heat shock protein family D member 1 (HSPD1), eukaryotic translation elongation factor 1 gamma, heterogeneous nuclear ribonucleoprotein A2/B1 (HNRNPA2B1), fibrinogen beta chain (FGB), Talin 1 (TLN 1), adaptor-related protein complex 2 subunit alpha-2 (AP2A2), serrated RNA effector molecule homolog (SRRT), apolipoprotein C3 (APOC3), and phosphoglucomutase 5 (PGM5). Fibrinogen beta chain is a key biomarker for CRLM.
Kim EK et al. [46] 2019 *Cancer Genomics Proteomics*	Fresh frozen tissue	2D-PAGE, MALDI-TOF MS	*n* = 5 CRLM Comparison: *n* = 5 synchronous primary CRC	Upregulation of serpin family A member 1 (SERPINA1), apolipoprotein AI (APOA1), intelectin 1 (ITLN1), desmin (DES), diazepam-binding inhibitor (DBI), succinate dehydrogenase complex flavoprotein subunit A (SDHA), and carbonic anhydrase 1 (CA1) in CRLM.
Kirana C et al. [47] 2019 *Clinical Proteomics*	Fresh frozen tissue	2D-DIGE, MALDI-TOF MS	*n* = 8 stage II CRC with CRLM within 5 years after surgery Comparison: *n* = 11 stage II CRC patients with no metastasis within 5 years after surgery	Upregulation of HLA class I histocompatibility antigen, B alpha chain (HLAB), A disintegrin and metalloproteinase with thrombospondin motifs 2 (ADAMTS2), latent-transforming growth factor beta-binding protein 3 (LTBP3), protein jagged-2 (JAG2) and nucleoside diphosphate kinase B (NME2) on tumour cells was associated with CRC progression and invasion, metastasis and CRC-specific survival.
Yuzhalin AE et al. [48] 2018 *Nature Communications*	Fresh frozen tissue	ECM enrichment, label-free, nano-LC-MS/MS	*n* = 5 CRLM Comparison: *n* = 5 matched normal liver, primary CRC and normal colon.	Increased amounts of citrullinated proteins in CRLM compared to normal liver. Primary CRC and normal colonic mucosa. Peptidylarginine deiminase 4 (PAD4)-driven citrullination of the extracellular matrix is essential for CRLM growth. Other upregulated proteins included versican (VCAN), metalloproteinase inhibitor 1 precursor (T1MP1), latent-transforming growth factor beta-binding protein (LTBP) 1–3, epithelial discoidin domain-containing receptor 1 (DDR1), and protein S100-A10 (S100A10).
Yang Q et al. [49] 2017 *Journal of Proteomics*	Fresh frozen tissue	1D and 2D-PAGE, nano-LC-MS/MS	*n* = 8 CRLM Comparison: *n* = 8 matched primary, CRLM and adjacent normal colon and liver tissues.	Olfactomedin 4 (OLFM4), CD11b/integrin alpha m (ITGAM) and integrin alpha-2 (ITGA2) significantly overexpressed in primary CRC and CRLM
Shen Z et al. [50] 2016 *Journal of Proteomics*	Fresh frozen tissue	Acetylated peptide enrichment, TMT labelling, LC-MS/MS	*n* = 3 CRLM Comparison: *n* = 3 matched primary CRC	HIST2H3AK19Ac and H2BLK121Ac were the acetylated histones most changed. Tropomyosin beta chain (TPM2), K152Ac and alcohol dehydrogenase 1B (ADH1B), K331Ac were the acetylated non-histones most altered in CRLM.
Naba et al. [51] 2014 *BMC Cancer*	Fresh frozen tissue	ECM enrichment, off-gel electrophoresis, LC-MS/MS	*n* = 3 CRLM Comparison: *n* = 3 matched primary CRC and normal colonic tissue	Hemopexin (HPX), osteopontin/secreted phospho-protein 1 (SPP1), cartilage oligomeric matrix protein (COMP), insulin-like growth factor-binding protein complex acid labile subunit (IGFALS), fibronectin type III domain-containing protein1 (FNDC1), bone morphogenetic protein 1 (BMP1) and complement C1q tumour necrosis factor-related protein 5 (C1QTNF5). Extracellular matrix protein signatures are potential tissue or serological biomarkers.
Turtoi A et al. [52] 2014 *Hepatology*	FFPE tissue	MALDI-MS imaging, nano-UPLC-qTOF MS	*n* = 8 CRLM Comparison: *n* = 8 normal liver, *n* = 3 matched primary CRC	High expression of latent-transforming growth factor beta-binding protein 2 (LTBP2) and transforming growth factor-beta-induced protein ig-h3 (TGFBI) were consistent features of CRLM and are absent in normal tissues.
Kirana et al. [53] 2012 *International Journal of Proteomics*	Fresh frozen tissue	2D-DIGE, MALDI-TOF MS	*n* = 8 CRLM Comparison: *n* = 8 matched primary CRC	Overexpression of cathepsin D (CTSD) in cells from the main tumour body showed significant correlation with subsequent distant metastasis and shorter cancer-specific survival.

**Table 2:** Studies on proteogenomics of colorectal cancer liver metastases.

Authors	Biospecimen	MS Technique	Sample Number with CRLM	Key Findings
Li C et al. [54] 2020 *Cancer Cell*	Fresh frozen tissue	Phosphopeptide enrichment, nano-LC-MS/MS	*n* = 43 Comparator: *n* = 146 primary CRC, adjacent normal colon and normal liver	Three CRC subtypes with distinct molecular signatures and clinical prognosis were defined using proteomic profiling. Phosphoproteomic pattern distinguishes metastatic from non-metastatic colorectal cancer.
Blank-Landeshammer B et al. [55] 2019 *Cancers (Basel)*	Fresh frozen tissue	Phosphopeptide enrichment, stable heavy isotope peptide labelling, nano-LC-MS/MS	*n* = 8 Comparator: *n* = 6 paired normal liver tissue	Low expression of actionable somatic mutations including KRASG12V can be predicted by precise quantitation of altered proteins such as SRPX2, S6K-alpha-5, GTPase KRas, PTBP1, ARL2, PPP1R14C and HAUS7
Ma YS. [56] 2019 *Molecular Therapy Oncolytics*	Fresh frozen tissue	Label-free nano-LC-MS/MS	*n* = 23 Comparator: *n* = 21 paired normal colorectal cancer tissue with or without liver metastasis	UQCR5 and FDFT1 were frequently overexpressed in the CRLM cohort and shown to have potential prognostic value. High expression of UQCR5 and was associated with worse overall survival and progression-free survival. High expression of FDFT1 was associated with better overall survival and progression-free survival.
Ma YS et al. [57] 2018 *Molecular Cancer*	Fresh frozen tissue	Nano-LC-MS/MS-based shotgun proteomics profiling	*n* = 23 Comparator: *n* = 21 non-metastatic CRC	Four CNV-mRNA-protein correlated proteins were associated with worse overall survival: HSP90AB1, COL1A2, FABP5 and BGN. Two single amino acid variants were associated with shorter overall and disease-free survival: MYH9 and CCT6A
Snoeren N et al. [58] 2013. *British Journal of Cancer*	Fresh frozen tissue	SDS-PAGE gel electrophoresis and in-gel digestion, label-free nano-LC-MS/MS	*n* = 10 <6 months to recurrence (early), *n* = 5 >24 months to recurrence (prolonged), *n* = 5	SERPINB5 which encodes for Maspin was the most upregulated (~2.1 times higher, *p* = 0.01) in patients with early recurrence compared to prolonged (>24 months) time to recurrence. Maspin was the only overlapping factor among 14 genes and 46 genes that showed a significant association with recurrence.
